# Studying Hepatitis Virus-Host Interactions in Patient Liver Biopsies

**DOI:** 10.3390/v14112490

**Published:** 2022-11-10

**Authors:** Aleksei Suslov, Markus H. Heim, Stefan Wieland

**Affiliations:** 1 Department of Biomedicine, University Hospital Basel, University of Basel, CH-4031 Basel, Switzerland; 2 Division of Gastroenterology and Hepatology, University Hospital Basel, CH-4031 Basel, Switzerland

**Keywords:** hepatitis B virus, hepatitis C virus, chronic infection, liver, liver biopsy

## Abstract

Infectious diseases are a major contributor to human suffering and the associated socioeconomic burden worldwide. A better understanding of human pathogen-host interactions is a prerequisite for the development of treatment strategies aimed at combatting human pathogen-induced diseases. Model systems that faithfully recapitulate the pathogen-host interactions in humans are critical to gain meaningful insight. Unfortunately, such model systems are not yet available for a number of pathogens. The strict tropism of the hepatitis B (HBV) and C (HCV) viruses for the human liver has made it difficult to study their virus-host interactions during the natural history of these infections. In this case, surplus liver biopsy tissue donated by patients provides an opportunity to obtain a snapshot of the phenomenological and molecular aspects of the human liver of chronically HCV or HBV-infected patients. In this review, we will briefly summarize our own efforts over the years to advance our knowledge of the virus-host interactions during the natural history of chronic HCV and HBV infection.

## 1. Introduction

**Figure 1:**
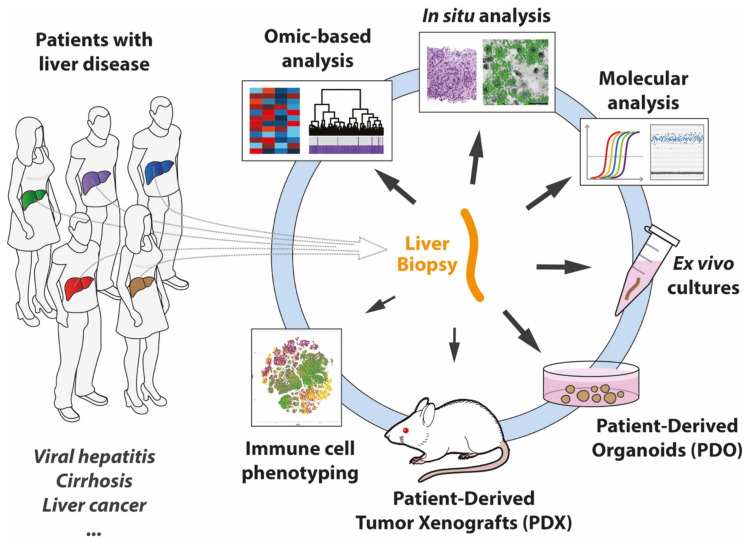
Liver biopsy tissue is the starting point for the study of liver diseases at the whole tissue and cellular level and the establishment of disease model systems.

Studying the human liver and its associated pathologies have remained challenging due to the lack of optimal model systems. This is particularly true for the study of hepatotropic virus infections. Primary human hepatocytes (PHH) have been considered the gold standard for a long time. However, their availability is limited, and variability between donors complicates experimentation. Most importantly, PHH can only be kept in culture for a relatively short time, thus limiting their usefulness for studying chronic liver diseases such as those associated with viral infections. Nevertheless, recent advances enabled PHH experimentation for several weeks rather than days [1]. In addition, alternative in vitro liver/hepatocyte model systems that promise to improve the experimental possibilities are under development [2,3]. Human liver chimeric mice have proven to be very valuable in vivo system for the study of infections with hepatotropic viruses [4]. A limitation of the chimeric mice is that they are immunodeficient and lack all human non-parenchymal cells, but efforts to introduce a human immune system are promising [4,5]. Chimpanzees provide a fully permissive system to study the pathogenesis of hepatotropic viruses, but their usage has been severely restricted for ethical reasons [6]. However, thanks to the generosity of patients that during routine liver biopsy sampling or in the context of a clinical study, donate a piece of liver tissue for science, we have the opportunity to obtain a glimpse into the human liver and the phenomenological and molecular aspects of different liver pathologies including those associated with hepatitis virus infections (Figure 1). For example, snap-frozen liver biopsy tissue is ideal for molecular profiling (i.e., analysis of viral and host nucleic acids and proteins).

Appropriately embedded and frozen tissue allows for nucleic acid analysis at the cellular level by highly specific and sensitive in situ hybridization [7,8]. Furthermore, fresh liver biopsy tissue can be used in different ways, including for short-term ex vivo experiments [9] and the establishment of liver-derived organoid cultures [10,11,12] or intrahepatic immune cell phenotyping [13,14]. Biopsies of liver tumors additionally allow for the establishment of patient-derived tumor organoids [11] and xenografts in mice [15]. Together these approaches facilitate the study of the underlying causes that lead to hepatocellular carcinoma (HCC) development in the liver and address the difficulties in achieving successful HCC therapy. HCC is the most common primary liver cancer and the second cause of cancer-related mortality worldwide [16]. HCC mainly arises in livers with pre-existing underlying diseases such as alcoholic liver disease, nonalcoholic steatohepatitis, and viral hepatitis. Among the latter, chronic infection with hepatitis B (HBV) or hepatitis C virus (HCV) represents a major risk factor for the development of hepatocellular carcinoma [16,17,18]. Thus, a better understanding of the virus-host interactions during chronic HBV and HCV infection has the potential to reveal mechanisms that could be therapeutically targeted with the aim of curing the infections and/or preventing disease progression to HCC. As indicated above, liver biopsies obtained from infected patients provide an opportunity to study the host-virus interactions in the liver that regulate chronic infection. In line with the topic of this special issue, this review is focused on the insight into hepatitis virus-host interactions we were able to gain from the study of liver biopsy tissue donated by patients in Switzerland.

## 2. Hepatitis Viruses

Hepatitis A, B, C, D, and E viruses are grouped together because they all target the liver and cause liver disease (i.e., hepatitis), even though they are phylogenetically unrelated. Hepatitis A and E viruses are predominantly transmitted by the fecal-oral route and mostly cause acute self-limited inflammatory liver disease and therefore do not present an immediate epidemiological threat [19].

HBV and HCV are transmitted via blood and often cause chronic infection (chronic hepatitis B (CHB) and chronic hepatitis C (CHC)), leading to gradually progressing liver disease [18,20]. Together these two viruses infect >5% of the human population and cause >1 million deaths every year due to complications of the virus-related chronic liver disease leading to cirrhosis and hepatocellular carcinoma [21,22]. Despite the availability of a protective vaccine for HBV and very efficient antiviral therapies against HCV, these viruses are still a major global health problem, especially in the developing world [18,21,22].

The Hepatitis D virus is a satellite virus that completely depends on HBV for its life cycle [23]. HDV co- or superinfection occurs only in 5–10% of HBV-infected individuals but results in an accelerated liver disease progression and is associated with poor prognosis [24,25]. 

Most people exposed to HCV (~80%) develop a chronic infection, while the rest undergo a self-limiting acute HCV infection typically lasting up to 12 weeks [18]. Diagnosis of acute HCV infection requires a positive serological HCV PCR test, as only about 25% of acute HCV infections become clinically apparent. 10–20% of chronically HCV-infected patients, however, develop chronic hepatitis that can progress to cirrhosis and lead to HCC over a period of 30 years [21].

In contrast, 90% of adults exposed to HBV undergo an acute, self-limiting infection. These infections can be symptomatic or asymptomatic [26]. Perinatal and early childhood transmissions of HBV, however, have a very high (~90%) propensity to become chronic and thus lead to the development of chronic liver disease that can progress to cirrhosis and HCC [22]. 

The outcome of HBV and HCV infections, as well as disease progression, is the result of specific virus-host interactions in the infected hepatocytes, between cells in the liver and in the periphery of infected patients [27,28]. Studying these interactions has been complicated by the fact that humans and chimpanzees are the only natural hosts for HBV and HCV [29]. Indeed, chimpanzees have been instrumental in studying the pathogenesis of HBV and HCV infections in the liver and periphery for a long time, but their usage is now severely restricted for ethical considerations [6]. In a few cases, accidental needlestick exposure to HCV or early detection of an HBV infection during routine screening provided an opportunity for longitudinal analysis of the virological and immunological aspects of HCV and HBV infections in patients [30,31,32]. Otherwise, studies in infected patients are typically cross-sectional in nature and often restricted to the periphery. Nevertheless, numerous studies have revealed that chronic HBV and HCV infection is associated with weak adaptive immune responses characterized by a T cell exhaustion phenotype [27,28,33]. However, the mechanisms leading to the impairment and ultimate failure of the innate and adaptive immune systems to clear these infections remain poorly understood [18,20,34]. In the following we review some aspects of the innate immune responses and virus host interactions that we studied using patient liver biopsies.

## 3. Innate Immune Response

The first line of defense of a host against pathogens is known as the innate immune response. Innate immune responses are not pathogen-specific but are triggered by molecules common to many pathogens (pathogen-associated molecular patterns; PAMPs) [35]. PAMPs are sensed by specific pattern-recognition receptors (PRRs) that are expressed in many cell types. PRR signaling induces a broad spectrum of responses, including the production of type I/III interferons (IFNs) and other cytokines that induce a wide range of genes with antiviral activity [35,36,37,38]. These innate immune responses are also crucial for subsequent activation of pathogen-specific adaptive immune responses involving T- and B-cells that typically clear the infection by killing the infected cells and preventing the spread of the virus with antibodies, respectively [38]. Not surprisingly, most pathogens, including viruses, have acquired defense mechanisms targeting the host’s innate immune responses [39]. This interplay between virus and host plays an important role in the outcome of an infection (i.e., acute resolving vs. chronic infection).

Because of the strict tropism of HBV and HCV for hepatocytes, it is primarily the liver microenvironment, together with the various liver sinusoidal cell populations, that determines the outcome of a hepatic infection [27]. Of note, the balance between immunity and tolerance is very strictly regulated in the liver in order to avoid overt organ damage due to strong immune activation by degradation products of pathogens transported from the gut to the liver [27]. This liver-specific immune regulation is likely contributing to the development of chronic hepatitis B and C infection.

## 4. Chronic HCV Infection

HCV is an enveloped positive (+) strand RNA virus belonging to the Flaviviridae family. HCV genomic RNA contains secondary structures and sequence elements that serve as PAMPs [40]. HCV replication is associated with the formation of “membranous web” structures in the infected hepatocyte, where the viral RNA-dependent RNA polymerase (RdRp) drives viral RNA synthesis. The replication process involves double-stranded (ds)RNA intermediates that again represent PAMPs [35,41]. Thus, it is not surprising that HCV infection induces a strong intrahepatic innate immune response as evidenced by strongly induced interferon-stimulated gene (ISG) expression in the liver of infected chimpanzees [42,43]. Despite the strong innate response to HCV, 70–80% of patients fail to clear the infection and develop CHC [18]. Interestingly, about half of the CHC patients maintain a type I/III IFN-induced gene expression signature in the liver [44,45]. This suggests that HCV not only overcomes the initial innate antiviral defenses of the host but that it can persist in the liver in the presence of a continuous innate immune response [42,46]. 

It is likely that viral persistence is at least in part facilitated by the immune evasion mechanisms of HCV. Indeed, the HCV NS3/4a protease has been shown to block IFN induction in vitro by cleavage of the mitochondrial antiviral-signaling (MAVS) adaptor protein [47]. We confirmed MAVS cleavage in the liver of CHC patients, but there was only a very weak negative correlation between the degree of MAVS cleavage and the level of ISG expression [48]. Thus, it could be speculated that HCV is sensed by non-parenchymal cells in the liver and that ISG expression is predominantly induced in uninfected hepatocytes. Using a highly sensitive duplex in situ hybridization (ISH) system, however, we were able to visualize HCV-infected cells in the liver of CHC patients and could demonstrate that the intrahepatic ISG induction is strongest in and around the HCV-infected cells [8]. This suggested that intrahepatic ISG expression is not driven by systemic interferon but rather by the HCV-infected hepatocyte in an autocrine and paracrine manner.

The question remained whether ISG expression was driven by type I or III IFNs. Interestingly, one of the ISGs expressed in the liver of CHC patients is ubiquitin-specific peptidase 18 (USP18) [49], a known interferon-inducible negative regulator of IFNα signaling [50]. Since USP18 does not interfere with IFNʎ signaling [51], it is likely that the ISG response observed in the liver of CHC patients is maintained by a type III IFN. Indeed, genetic and molecular studies linked intrahepatic ISG expression in CHC patients with IFNʎ4, a new member of the type III IFN family [52,53,54,55]. Specifically, we found that the fully active IFNʎ4 variant was associated with the strongest ISG expression in the liver of CHC patients [55]. In vitro studies demonstrated that the IFNʎ4 protein is poorly secreted but that it is a highly potent ISG inducer [10,56]. These characteristics of IFNʎ4 could explain the apparent autocrine and paracrine IFN activity in the liver of CHC patients [8].

Paradoxically, however, genome-wide association studies revealed that genetic variants encoding functional IFNʎ4 are associated with reduced spontaneous clearance of HCV [52,53,54,55]. Using cell culture systems, we could recently demonstrate that intracellular IFNʎ4 induces endoplasmic reticulum (ER) stress resulting in impaired HCV antigen expression and attenuation of HCV-specific T cell responses [10]. Thus, these results could explain the reduced HCV clearance rate in the presence of functional IFNʎ4. 

Before the advent of highly potent direct-acting antivirals (DAAs) that allow the curing of chronic HCV infection, IFNα treatment has been the mainstay of HCV antiviral therapy [57]. Interestingly, and contrary to the expectation, we observed that a strong intrahepatic endogenous ISG expression in CHC patients was very tightly associated with a non-response to IFNα therapy [45,58]. In fact, a strong up-regulation of ISGs upon IFNα treatment and rapid virological response (i.e., serum HCV RNA undetectable after 4 weeks of treatment) was only observed in patients presenting with low or absent endogenous ISG expression [45,58]. In contrast, in patients with high endogenous ISG expression (i.e., pre-activated endogenous IFN system), IFNα therapy failed to further up-regulate ISG expression and clear the virus [45,58]. Presumably, IFNα signaling is blocked in the liver of these patients because of the preexisting expression of the negative regulator USP18 [59].

In a different study, we compared the intrahepatic ISG expression profile induced by IFNα therapy in CHC patients without endogenous ISG expression with that of CHC patients with a persistent innate immune response in the liver [60]. There were no qualitative differences between the IFNα induced ISG profile and the persistent innate immune response [60]. Quantitatively, however, the level of ISG expression in response to IFNα therapy was much higher than that observed in CHC patients with endogenous ISG expression [60]. These results suggest that the persistent innate immune response in CHC is not strong enough to effectively clear the virus.

In the same study, we could also investigate whether HCV infection led to changes in the intrahepatic transcriptional profile that are not associated with the innate immune response. Specifically, we compared the intrahepatic transcriptome of CHC patients lacking an activated endogenous IFN system with that of uninfected control patients [60]. These studies revealed that transcriptomic changes in the HCV-infected liver are predominantly associated with immune cell functions but not with hepatocyte-intrinsic pathways [60]. Since the global liver transcriptome was analyzed in this study, it is possible that transcriptional alterations in infected hepatocytes were masked by unchanged gene expression in uninfected hepatocytes and non-parenchymal liver cells. Future studies using single-cell sequencing could address this question and could also allow for a detailed analysis of the contribution of other liver cell populations to the transcriptional landscape in the HCV-infected liver.

## 5. Chronic HBV Infection

HBV is a small, enveloped DNA virus belonging to the Hepadnaviridae family [61]. Upon infection of the hepatocyte, the viral genome is converted into a covalently closed circular (ccc)DNA episome that persists in the nucleus. cccDNA is transcribed by the host RNA polymerase machinery into a set of overlapping capped and polyadenylated co-terminal mRNAs that are exported into the cytoplasm of the hepatocyte [61]. The pregenomic (pg)RNA, together with the viral polymerase (RT-Pol), is sequestered in the viral core particle within which the pgRNA is reverse transcribed, and the partially double-stranded (ds)DNA genome is synthesized by the RT-Pol. dsDNA-containing capsids are eventually enveloped with cellular membranes containing the viral envelope proteins and released from the cell as infectious virions [61].

Clinically, the natural history of CHB has been divided into five phases based on virological and disease parameters [62]. HBeAg-positive chronic infection (EPCI), characterized by high viremia and antigenemia in the absence of any disease, is followed by the HBeAg-positive chronic hepatitis B (EPCHB) phase with elevated alanine aminotransferase (ALT) levels indicating the presence of an active antiviral immune response. The subsequent loss of HBeAg in the serum leads into the HBeAg-negative phase, which is divided into HBeAg-negative chronic hepatitis B (ENCHB) and HBeAg-negative chronic infection (ENCI). In many cases, HBeAg-loss has been ascribed to mutations in the precore and/or basal core promoter regions in the viral genome that lead to low or absent expression of HBeAg [63]. During ENCI, the virus remains undetectable in the serum or contained at very low levels, and there is no evidence of liver disease. ENCHB, on the other hand, is associated with persistent liver disease and fluctuating but moderate viral loads. Lastly, the HBsAg-negative stage is defined by the disappearance of serum HBsAg and the absence of liver disease, with or without the appearance of anti-HBs antibodies (i.e., HBsAg seroconversion).

Surprisingly and in contrast to HCV, acute HBV infection does not trigger an innate IFN response in the liver of chimpanzees [64] which likely contributes to the development of CHB [64,65]. Whether the absence of a cellular innate immune response during HBV infection is due to HBV being invisible to the cell-intrinsic innate sensing machinery (i.e., acting as a stealth virus) or whether HBV actively suppresses innate immune responses remained controversial [65]. To address this question, we developed a short-term ex vivo culture system for biopsy pieces that enabled the investigation of pathogen-induced cellular innate immune responses in intact human liver tissue [9]. Using liver biopsies obtained from CHB patients across all disease phases and uninfected control patients, we found that neither induction of innate immune response nor IFN signaling was impaired in HBV-infected hepatocytes, indicating that HBV is not suppressing the innate immune system in the human liver [9]. Furthermore, these experiments demonstrated a complete lack of an intrahepatic innate immune response signature during all phases of CHB [9]. Together, these studies confirmed the hypothesis that HBV is invisible to the innate sensing machinery and behaves like a “stealth” virus in this regard [9]. This “invisibility” likely reflects the replication strategy of HBV, which retains the transcriptional template in the nucleus, involves the production of capped and polyadenylated viral mRNAs that resemble the structure of normal cellular transcripts, and sequesters its replicating genome within viral capsid particles in the cytoplasm [64]. Together with similar observations in state-of-the-art cell culture systems [66,67], these findings suggest that the evaluation of specific innate response inducers as therapeutic interventions for CHB might be warranted [65].

The molecular mechanisms that control intrahepatic HBV gene expression and replication in and during the transition between the different disease phases of CHB are not yet fully understood. Liver biopsy samples are required to study these mechanisms because current model systems cannot fully recapitulate the natural history of CHB [1,2,3,4,5]. We recently began to identify the steps in the viral life cycle that are controlled during different disease phases in the liver of CHB patients [68]. Specifically, we found that cccDNA transcriptional activity is strongly suppressed in the ENCI stage of CHB, while the replication efficiency was similar to that in the liver of HBeAg+ (EPCI and EPCHB) patients [68]. Interestingly, we also found that the HBV replication efficiency was specifically suppressed in the liver of a subgroup of ENCHB patients compared to that in the ENCI and HBeAg+ cohort [68]. Together, these studies identified specific patterns of viral control in different disease stages of CHB. Furthermore, they revealed that distinct mechanisms of viral control can be active in the liver of patients clinically grouped into the same disease phase [68].

HBV integration is not an obligatory step in the HBV viral life cycle. Mostly, it results from a failure of proper template-switching during viral replication known to produce double-stranded linear DNA (dslDNA), the presumed form of viral DNA for HBV integration [69]. Importantly, the structure of the integrated dslDNA renders it replication incompetent. However, integrated HBV DNA still allows for transcription of the viral RNAs encoding for the viral envelope proteins [70]. Therefore, HBV integration has recently gained renewed attention as it can have a direct impact on the development and efficacy of new antiviral therapies [71]. Interestingly, there are patients in the ENCI phase of CHB that present with >90% HBsAg-positive hepatocytes in the liver despite very low or negative viremia [72]. Using a combination of next-generation sequencing, in situ hybridization, and molecular biology approaches, we were able to demonstrate that HBV integration rather than cccDNA-driven transcription was responsible for HBsAg expression in these patients. These findings imply that transcriptionally active HBV integration can extend to the entire liver and could account for most of the HBsAg production in some CHB patients [72]. In such patients, HBsAg loss might not be an ideal marker for the resolution of HBV infection.

## 6. Concluding Remarks

Our studies on HBV and HCV virus-host interactions summarized in this review represent only a small part of the large body of similar studies that have been performed over many years. Liver biopsy tissue has proven to be an invaluable resource for many of these studies. Importantly, however, there is no doubt that it will be the integration of the results obtained with all the different model systems that will ultimately lead to successful therapy of viral hepatitis infections and the associated liver pathologies.

## Data Availability

Not applicable.
